# A platinum black-modified microelectrode for in situ olanzapine detection in microliter volumes of undiluted serum

**DOI:** 10.1007/s00702-019-02139-0

**Published:** 2020-01-06

**Authors:** Rajendra P. Shukla, Robert H. Belmaker, Yuly Bersudsky, Hadar Ben-Yoav

**Affiliations:** 1 Nanobioelectronics Laboratory (NBEL), Department of Biomedical Engineering and Ilse Katz Institute of Nanoscale Science and Technology, Ben-Gurion University of the Negev, 8410501 Beer-Sheva, Israel; 2 Department of Psychiatry, Mental Health Center, Ben-Gurion University of the Negev, 8410501 Beer-Sheva, Israel

**Keywords:** Antipsychotic olanzapine, Serum, Electrochemical sensors, Point-of-care testing, Therapeutic drug monitoring, Reduced graphene oxide

## Abstract

Olanzapine is a thienobenzodiazepine compound. It is one of the newer types of antipsychotic drugs used in the treatment of schizophrenia and other psychotic disorders. Several methods have been reported for analyzing olanzapine in its pure form or combined with other drugs and in biological fluids. These methods include high-performance liquid chromatography and liquid chromatography-tandem mass spectroscopy. Although many of the reported methods are accurate and sensitive, they require the use of sophisticated equipment, lack in situ analysis, and require expensive reagents. Moreover, several of these methods are cumbersome, require prolonged sample pretreatment, strict control of pH, and long reaction times. Here we present the development of a miniaturized electrochemical sensor that will enable minimally invasive, real-time, and in situ monitoring of olanzapine levels in microliter volumes of serum samples. For this purpose, we modified a microfabricated microelectrode with a platinum black film to increase the electrocatalytic activity of the microelectrode towards olanzapine oxidation; this improved the overall selectivity and sensitivity of the sensor. We observed in recorded voltammograms the anodic current dose response characteristics in microliter volumes of olanzapine-spiked serum samples that resulted in a limit of detection of 28.6 ± 1.3 nM and a sensitivity of 0.14 ± 0.02 µA/cm^2^ nM. Importantly, the platinum black-modified microelectrode exhibited a limit of detection that is below the clinical threshold (65–130 nM). Further miniaturizing and integrating such sensors into point-of-care devices provide real-time monitoring of olanzapine blood levels; this will enable treatment teams to receive feedback and administer adjustable olanzapine therapy.

## Introduction

Olanzapine (OLZ) is the most commonly used second-generation antipsychotic medication approved by the FDA for Schizophrenia treatment. Patients with mental health problems are advised to take this medicine once a day; the doses range from 5 to 10 mg, depending on the patient’s illness condition (Lin et al. 2017). Therapeutic drug monitoring of this drug is very important in order to improve the efficacy of the treatment (Lu et al. 2018). Current state-of-the art technology, which is used to monitor Olanzapine levels in patients, includes high-performance liquid chromatography and liquid chromatography-tandem mass spectroscopy. Unfortunately, these methods require frequent and invasive blood draws from patients. This is a painful process that requires trained personnel to deal with sophisticated instruments, thus increasing the cost of treatment. In addition, it is a time-consuming process that includes admitting patients and obtaining a report from the central labs (Ni et al. 2018; Wu et al. 2019). Overall, it is an added burden on the patients in terms of time and money.

Olanzapine is a redox active molecule (it can donate and receive electrons); therefore, electrochemical sensors can provide an alternate solution to the current state-of-the-art analytical methods due to their simple design and easy manufacturing process as well as their high sensitivity and cost effectiveness, compared with the currently available analytical tools for analyzing redox molecules (Merli et al. 2012). Electrochemical sensors have been previously used to detect OLZ; however, they suffer from several challenges. Currently no available sensor can monitor the OLZ blood levels in situ and at the point of care. El-Shal reported the electrochemical sensing of OLZ using a glassy carbon electrode in Britton-Robinson buffer (pH 2.0) solution; the limit of detection (LOD) was 1 × 10^–8^ M (El-Shal 2013). However, El-Shal’s research did not detect OLZ in biological samples, such as serum. Arvand and Palizkar reported on the electrochemical detection of OLZ using an amine-functionalized TiO_2_/multi-walled carbon nanotube consisting of a composite-modified glassy carbon platform (Arvand and Palizkar 2013). They reported a LOD value of 90 nM in phosphate buffered saline (0.1 M, pH 5) and applied the same platform to recover OLZ in diluted serum samples in same buffer solution. The effect of solution pH on OLZ oxidation was studied; it was found that the solution pH significantly influences the oxidation peak current. The oxidation peak current was maximum at pH 5.0; however, it decreased at higher pH values due to proton deficiency. As a result, pH 5.0 was selected for this study. Ahmed et al. demonstrated OLZ detection with a modified carbon paste sensor electrode incorporating gold nanoparticles and glutamine in a micellar medium (Ahmed et al. 2015). They reported an LOD value of 3.58 × 10^–9^ M in Britton-Robinson buffer (pH 7.0) and demonstrated the recovery of OLZ in urine samples. However, this study did not show how interfering species affect the detection of OLZ in biological samples within their therapeutic range. For a point-of-care device, a smaller sample volume (a few microliters) is also required. Arvand et al. reported an electrochemical sensor based on Fe_3_O_4_@Ag core/shell magnetic nanoparticles—a modified carbon paste electrode that was used to investigate the electrochemical behavior of olanzapine in acetate buffer (pH 4.3) (Arvand et al. 2015). They reported an LOD value of 0.0018 μM in acetate buffer and used the same sensor to recover OLZ from tablets and in diluted serum samples from schizophrenia patients. However, this study also did not demonstrate real-time and in situ analysis of OLZ in microliter volume samples at the point of care. Recently, the group of Rouhani et al. demonstrated OLZ detection in diluted serum samples using a carbon paste electrode.(Rouhani and Soleymanpour 2019) Although the results of these studies are promising, they did not demonstrate an improved sensing performance in micro-systems integrated with microelectrodes using micro-liter sample volumes. Several challenges are associated with microscale analytical devices, including low signal-to-background ratios as well as physical and chemical effects (e.g., capillary forces, surface roughness, and chemical interactions of the construction material with the reaction processes). In addition, the sensitivity and specificity of these micro-systems are reduced when dealing with biological fluids (e.g., urine and serum) due to the presence of other electro-active species in the solution. These challenges of using an analytical device limit the ability to detect low levels of drugs in biological fluids. However, by utilizing a miniaturized lab-on-a-chip electrochemical micro-system, schizophrenia treatment outcomes can be improved.

**Fig. 1:**
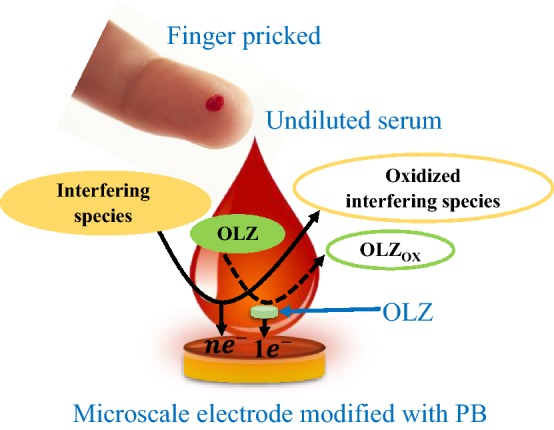
Scheme of olanzapine detection in microliter volumes of undiluted serum samples using a platinum black-modified microelectrode

Here we report on a platinum black (PB)—modified microelectrode for the in situ detection of OLZ in microliter volumes of undiluted serum samples (Fig. 1). Platinum black film has been used for electrochemical sensing applications because of its high conductivity and electrocatalytic activity (Li et al. 2013; Seo et al. 2008). The platinum black-modified microelectrode enhances the effective surface area, resulting in significantly increased OLZ oxidation current. The increased oxidation process of OLZ results in better sensitivity and improved LOD values in the buffer as well as in undiluted serum samples. Interestingly, a bare Au microelectrode was unable to differentiate between different OLZ concentrations, whereas a PB-modified microelectrode exhibited a positive dose response with a high background. More specifically, the PB-modified microelectrode exhibited a sensitivity of 0.14 ± 0.02 µA/cm^2^ nM and an LOD value of 28.6 ± 1.3 nM. The LOD value is well below the clinical threshold. This proof-of-concept study can be further miniaturized to point-of-care devices and integrated into wearable, handheld devices for better management of schizophrenia treatment.

## Materials and methods

### Chemicals

The following materials have been used without further purification: OLZ (catalog number: 1478301, Merck), Dihydrogen hexachloroplatinate (IV) hexahydrate (chloroplatinic acid; catalog number: 011051, Alfa aesar), 99% Lead(II) acetate trihydrate (lead acetate; catalog number; A11746, Alfa Aesar), di-sodium hydrogen phosphate dihydrate (catalog number: 1.06580.1000, Merck), sodium dihydrogen phosphate dihydrate (catalog number: 1.06342.0250, Merck), sodium chloride (catalog number: 1.06404.1000, Merck), potassium hexacyanoferrate(II) trihydrate (‘Ferrocyanide’, catalog number: 1.04984.0100, Merck), potassium hexacyanoferrate(III) (‘Ferricyanide’, catalog number: 1.04973.0100, Merck), acetone Find Bio-Lab, Ltd.), 2-propanol (catalog number: 001626052100, Bio-Lab, Ltd.) and hydrochloric acid 32% (catalog number: 000846050100, Bio-Lab, Ltd.). Ultra-pure water (> 18 MΩ) was obtained from a Super Q water system (Millipore). All prepared solutions were diluted in PBS (10 mM, pH 7.4) solution. For all the electrochemical tests, a three-electrode electrochemical cell, consisting of an Ag/AgCl reference electrode (a metal wire coated with Ag/AgCl ink; catalogue number: 011464, BAS, Inc.), a ring platinum counter electrode (catalogue number: 012961, ALS Co., Ltd.), and microfabricated microelectrodes as working electrodes were used.

### Preparation of buffered samples

A stock solution of 10 mg/ml OLZ was prepared in 2-propanol and stored at − 20 °C. OLZ standard solutions (100 µl each) of 20, 50, 90, 120, and 150 nM were prepared by diluting the 10 mg/ml OLZ stock solution with PBS (10 mM, pH 7.4).

### Preparation of undiluted serum samples

Serum samples were collected from a healthy 38-year-old adult volunteer. To prepare the serum samples, blood samples were collected in 15 ml tubes (BD Vacutainer® SSTTM II Advance, Fisher Scientific, Ltd.) and left for 30 min to allow the blood to clot. The clotted blood was centrifuged (1200 rpm for 10 min), and the supernatant was collected and stored in a new 15 mL tube. Following another centrifugation step (1200 rpm for 10 min), the supernatant was collected and 0.5 mL aliquots were stored in 1.5 mL Eppendorf tubes at − 20 °C. OLZ standard solutions (100 µl each) of 20, 50, 90, 120, and 150 nM were prepared by diluting the 10 mg/ml OLZ stock solution with an undiluted serum sample. Ethical approval was obtained from the Ben-Gurion University of the Negev (BGU) Human Subjects Research Committee.

### Microfabrication of gold microelectrodes

**Fig. 2:**
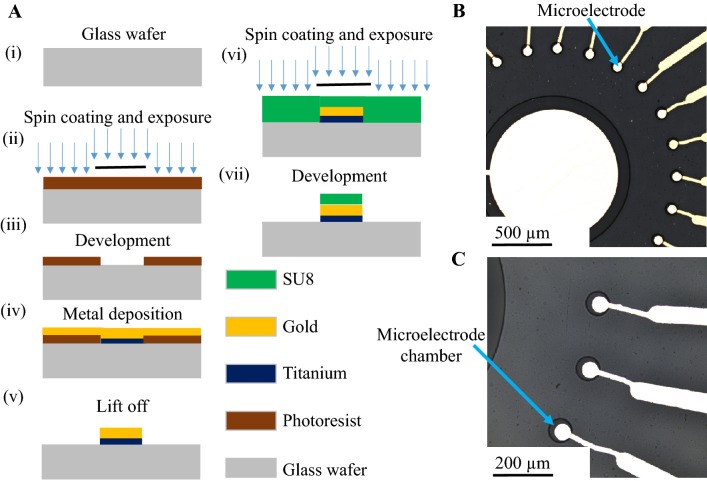
**a** Microfabrication of gold microelectrodes: (i) a cleaning glass wafer, (ii) the spin coating of the photoresist followed by exposure using the mask aligner, (iii) a developing patterned wafer in 726MIF developer solution, (iv) Ti deposition followed by Au onto the patterned photoresist, (v) metal lift off in acetone solution resulting in the Ti/Au microelectrode patterns, (vi) the spin coating SU8 negative photoresist and exposure using the mask aligner for defining the microelectrode chamber, and (v) the development of the SU8 patterned wafer in PGMA developer to define the chamber. **b** A low-resolution optical image of the microelectrodes and **c** a high-resolution optical image of the microelectrodes showing the well-defined SU8 microchambers

A borosilicate glass substrate was cleaned with acetone, isopropanol, and deionized (DI) water and then dried with nitrogen gas. The photoresist (AZ 5214E) coating process starts with spinning the wafer with the photoresist at 2200 RPM for 12 s, followed by a soft bake on a contact hot plate at 110 °C for 2 min and 30 s. Next, the electrode patterns were transferred from the mask using a hard contact of 7.6 mW/cm^2^ for 65 s using a mask aligner (Karl Suss Mask Aligner MA6). The exposed wafer was then developed in AZ 726 MIF developer for 6 min, followed by rinsing in DI water for 5 min. Next, 20 nm of titanium and 200 nm of gold were deposited using the E-gun deposition system. The wafer was then transferred to a beaker with acetone solution for a lift off process that resulted in the Au/Ti microelectrode patterns on a glass substrate. The wafer was again rinsed in the DI water for 1 min to remove any residue from the wafer. SU8-3005 was used to define the microelectrode chamber; this allows cleaning the microelectrode with an AMI (acetone, methanol, and isopropanol) solution without destroying the chamber before using it. The process starts with spin coating SU8-3005 at 3000 RPM for 30 s, followed by a soft bake on a contact hot plate at 95 °C for 15 min. Next, the photoresist was exposed to light through the electrodes’ mask using a hard contact of 7.6 mW/cm^2^ for 50 s at a Mask Aligner (MA6, SUSS MicroTec). Post Exposer Bake (PEB) for 5 min at 95 °C was used, since this is a negative photoresist and the wafer was cooled down to room temperature. The exposed wafer was then developed in PGMA ERB developer solution for 8 min and washed in isopropanol solution for 10 s. The hard bake on a contact hot plate at 150 °C for 5 min was carried out to remove any hydration on the substrate, and oxygen plasma cleaning (30 W, 500 mTorr, 2 min, 3 sccm) was used after the hard bake to remove any residues or impurities on the substrate. The wafer was then coated with photoresist before dicing it into chips using a Dicing saw (ADT-7100). All chips were cleaned with AMI solution before use. The microelectrode microfabrication flow process is shown in Fig. 2.

### Preparing platinum black deposition solution

Platinum black deposition solution was prepared by mixing 1% chloroplatinic acid and 0.05% lead acetate in DI water. The mixture was then stirred and 0.0025% of 32% hydrochloric acid was added to the mixture (Stanca et al. 2017). The prepared solution was covered with aluminum foil and stored at room temperature.

### Platinum black electrodeposition onto microscale electrodes

Before the gold microelectrode was modified, the chip was cleaned with AMI solution, followed by rinsing in DI water. The chip was then dried with nitrogen. The chronopotentiometry technique (current density = 4.8 mA/cm^2^, time = 5 min) was used modify the gold microelectrode.

### Surface characterization

The PB-modified microelectrode was visually inspected using an optical microscope; then the morphology of the PB surface was observed using a high-resolution scanning electron microscope (HRSEM; JSM-7400F-JEOL). The thickness of the PB film was characterized using Dektak profiler (Veeco Dektak-8).

### Electrochemical characterization

PB-modified electrodes were electrochemically characterized in 10 mM PBS (pH 7.4) and 5 mM ferricyanide/ferrocyanide solution. Cyclic voltammetry (CV; *E*_i_ = 0 V, *E*_1_ = − 0.2 V, *E*_2_ = 0.65 V, scan rate = 0.1 V/s, number of scans = 2) was used for testing the electrochemical activity.

### Olanzapine analysis in buffered and undiluted serum samples

The electrochemical detection performance of OLZ was tested in both buffered and undiluted serum samples with a sensor array. The Differential Pulse Voltammetry technique (DPV; a potential sweep between − 0.1 and 0.6 V, a potential increment of 0.05 V, an amplitude of 0.05 V, a pulse width of 0.009 s, a step height of 0.005 V, and a step time of 0.1 s) was used to obtain the dose response of OLZ; then the dose response plot was used to calculate the LOD and sensitivity. The slope of the linear regression of the dose response plot gives the sensitivity, whereas LOD is calculated as 3 standard deviations of the background signal. The repeatability of the PB-modified microelectrode was tested for detecting 150 nM OLZ-spiked undiluted serum samples. For this experiment, two microelectrodes were modified and the measurement was repeated 10 times for both microelectrodes. The PB-modified microelectrode was cleaned in between the measurements with PBS and DI water, followed by gently soaking the liquid with fine wipes.

## Results and discussion

### Platinum black-modified microelectrodes: modification and characterization

#### Microelectrode modification with platinum black and its electrochemical characterization

**Fig. 3:**
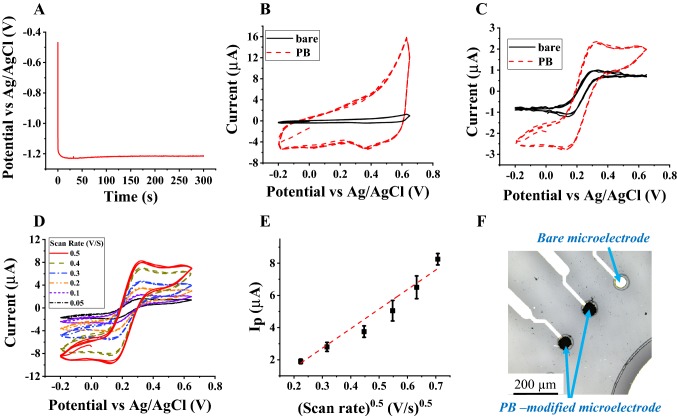
**a** Electrodeposition of PB on a gold microelectrode using Chronopotentiometry, **b** CVs measured in 10 mM PBS solution, and **c** in 5 mM ferrocyanide/ferricyanide at a scan rate of 0.1 V/s using a bare (black solid) and a PB-modified microelectrode (red dashes), **d** CVs measured at scan rates of 0.5 (solid red), 0.4 (dark yellow dashes), 0.3 (blue dashed dots), 0.2 (orange dashed dots), 0.1 (violet short dashes), and 0.05 (short black dashed dots) V/s by using a PB-modified microelectrode, **e** peak current vs the square root of the scan rate, and **f** an optical image of the PB-modified Au microelectrode

In order to selectively electrodeposit PB on the Au microelectrodes, the working microelectrode portions were immersed in the electrodeposition solution and connected accordingly. A constant cathodic current density of 4.8 mA/cm^2^ was applied to the working microelectrode for 5 min against a ring platinum counter electrode. The PB-modified Au microelectrode was then cleaned with DI water and dried with nitrogen. Figure 3a shows the potential versus the time characteristics during the electrodeposition. The electrochemical response of the modified microelectrode was tested in 10 mM PBS solution, followed by 5 mM ferrocyanide/ferricyanide solution. Figure 3b shows the CVs of the bare and the PB-modified microelectrode in buffered solution; this indicates the background signal (capacitive current). The PB-modified microelectrode exhibits a higher capacitive current because of its higher surface area, compared with the bare Au microelectrode. Figure 3c shows the CVs of the bare and PB-modified microelectrode in 5 mM ferrocyanide/ferricyanide. The electrochemical response in a 5 mM ferrocyanide/ferricyanide redox couple indicates that there was a higher oxidation current for the PB-modified microelectrode, compared with the bare one due to its high effective surface area.

We also calculated the effective surface area of the PB-modified microelectrodes using CV at different scan rates of 0.5, 0.4, 0.3, 0.2, 0.1, and 0.05 V/s in a 5 mM ferrocyanide/ferricyanide solution. The potential was scanned from − 0.2 to 0.65 V for 2 cycles. The oxidation current peak for the PB-modified microelectrode was plotted against the square root of the scan rate, and the effective surface area of the modified microelectrode was calculated (Fig. 3e).

We calculated the effective surface area of the modified electrode by using the Randles–Sevcik relationship in Eq. 1 (Bard and Faulkner 2001):

1 $$ I_{{\text{p}}} = 0.4463\left( {F^{3} /RT} \right)^{1/2} n^{3/2} A_{{{\text{eff}}}} D^{1/2} C^{*} \nu^{1/2} , $$

where *I*_p_ [A] is the peak current, *F* [C/mol] is the Faraday constant, *R* [J/mol K] is the universal gas constant, *T* [K] is the absolute temperature, *n* is the number of moles of electrons transferred in the cell reaction (*n* = 1 for a ferrocyanide/ferricyanide redox reaction), *A*_eff_ [cm^2^] is the active surface area of the electrode, *D* [cm^2^/s] is the diffusion coefficient of the electro-active species [0.72 × 10^–5^ cm^2^/s for ferricyanide and 0.67 × 10^–5^ cm^2^/s for ferrocyanide (Konopka and McDuffie 1970)], C* [mol/cm^3^] is the bulk concentration of the electro-active species, and *v* [V/s] is the linear potential scan rate.

The calculated *A*_eff_ value of the PB-modified microelectrode was found to be 3.5 × 10^–3^ cm^2^ ± 0.1 × 10^–3^ cm^2^, which is 4.5 times higher than that of the bare Au microelectrode (the *A*_eff_ value of the Au microelectrode calculated from the mask design is 7.8 × 10^–4^ ± 0.2 × 10^–4^ cm^2^). The higher surface area of the PB-modified microelectrode is due to the non-homogenous nature and the roughness of the PB material and is equivalent to 3 layers of PB (Wayu et al. 2016). Lastly, the optical image of the PB-modified microelectrode was recorded for visualizing the modification. Figure 3f shows the PB-modified and bare Au microelectrodes. We noted that despite the assumption of linearity in Fig. 3e, the curve exhibited some non-linearity. The non-linearity can be due to the different conditions used to derive Eq. (1) and the experimental data. For example, Eq. (1) was calculated, based on the assumption of semi-infinite linear diffusion. However, we recorded electrochemical signals from a sample drop that can contribute to a non-linear restricted diffusion element due to the limited diffusion attributed to the drop’s volume. Moreover, Eq. (1) was calculated for macro-scale electrodes under equilibrium conditions, whereas we used microelectrodes that increase the impact of the kinetic parameters (the electron transfer rate).

#### Surface characterization

**Fig. 4:**
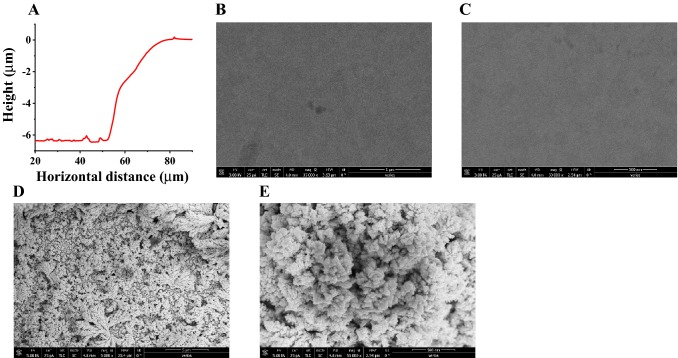
**a** Thickness of the PB film using the Stylus profiler, a low and high magnification micrograph of the bare **b** low, and **c** high magnification, and a PB-modified microelectrode **d** low, and **e** high magnification

The thickness of the PB-modified microelectrode was measured using the Dektak profiler, which showed a thickness of 6.5 µm (Fig. 4a). The PB-modified microelectrode was characterized using HRSEM to visualize the presence of PB on the Au microelectrodes. Figure 4b, c shows the low- and high-resolution micrographs of the bare gold microelectrode; Fig. 4d, e shows the surface of the PB-modified microelectrode, which is indicative of the presence of PB on the Au microelectrodes.

### Olanzapine sensing performance using a platinum black-modified microelectrode

**Fig. 5:**
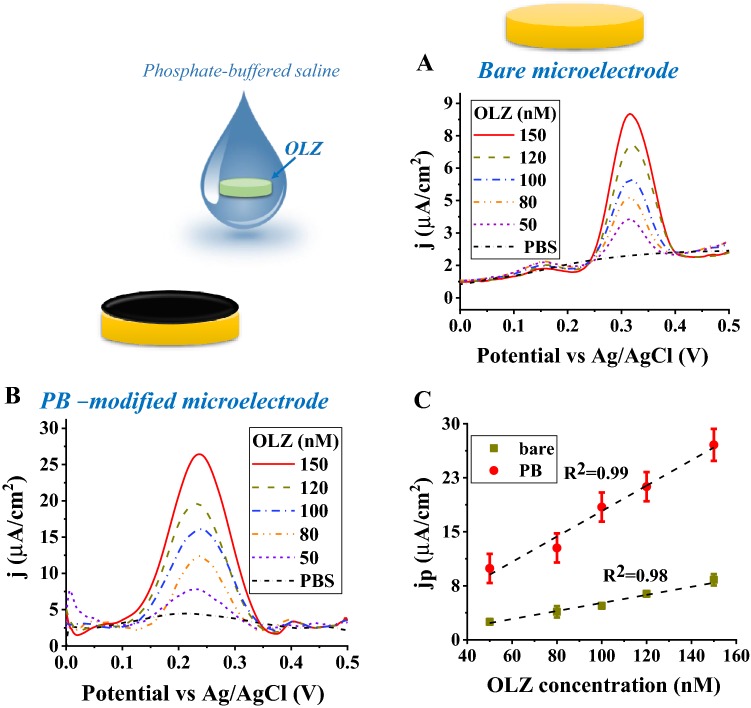
OLZ detection in PBS samples. DPVs of 150 (solid red), 120 (dark yellow dashes), 100 (blue dashed dots), 80 (orange dashed dots), 50 (blue short dashes), and PBS (short black dashed dots) nM OLZ concentrations using a **a** bare and **b** a PB-modified microelectrode. **c** Dose response plot for bare (dark yellow rectangles) and a PB-modified microelectrode (red circles)

Olanzapine sensing performance for the bare and the PB-modified microelectrode was tested in 20 µl of buffered samples. Differential pulse voltammetry was used to record the OLZ oxidation for different concentrations. A positive dose response was observed for the bare (Fig. 5a) and the PB-modified microelectrodes (Fig. 5b). The linear regression analysis of the positive dose response plot was used to calculate the sensitivity and the LOD (Eqs. 2 and 3).

*Bare microelectrode *(*R*^2^ = 0.97; LOD = 80.0 ± 13.3 nM):

2 $$ j_{{\text{anodic peak}}} = \left( {0.030 \pm 0.005 \,\,\upmu {\text{A}}/{\text{cm}}^{2} {\text{nM}}} \right)\left[ {{\text{OLZ}}} \right] + \left( {1.6 \pm 0.8 \,\,\upmu {\text{A}}/{\text{cm}}^{2} } \right) $$

*PB-modified microelectrode* (*R*^2^ = 0.98; LOD = 15.6 ± 0.8 nM):

3 $$ j_{{\text{anodic peak}}} = \left( {0.20 \pm 0.01 \,\,\upmu {\text{A}}/{\text{cm}}^{2} {\text{nM}}} \right)\left[ {{\text{OLZ}}} \right] + \left( {3.80 \pm 1.04\,\, \upmu {\text{A}}/{\text{cm}}^{2} } \right) $$

The dose response plot resulted in a sensitivity of 0.030 ± 0.005 µA/cm^2^ nM and 0.20 ± 0.01 µA/cm^2^ nM, as well as an LOD value of 80.0 ± 13.3 nM and 15.6 ± 0.8 nM for the bare and PB-modified microelectrodes, respectively. The PB-modified microelectrode is 6.7 times more sensitive than the bare one and has 5.1 times better sensing performance in terms of LOD, compared with the bare microelectrode. The improved sensing performance is due to the high surface area of the PB-modified microelectrode.

### Olanzapine detection performance in undiluted serum using a platinum black-modified microelectrode

**Fig. 6:**
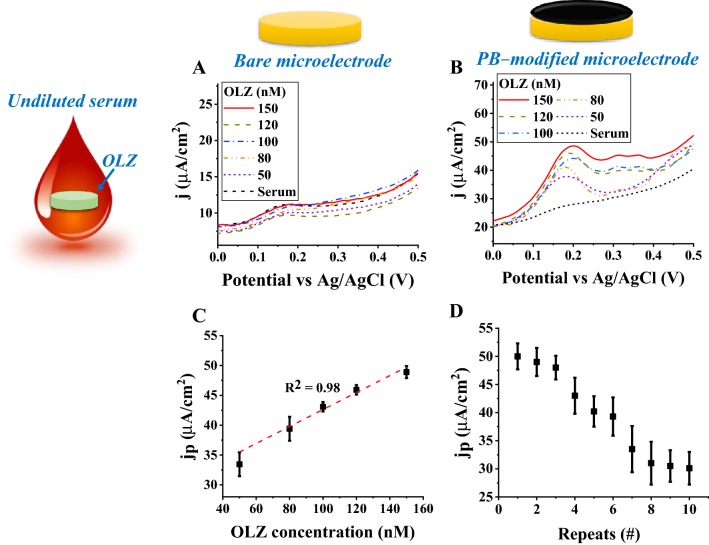
OLZ detection in undiluted serum samples. DPVs of 150 (solid red), 120 (dark yellow dashes), 100 (blue dashed dots), 80 (orange dashed dots), 50 (blue short dashes), and PBS (black short dashed dots) nM OLZ concentrations using a **a** bare and **b** PB-modified microelectrodes. **c** Dose response plot for a PB-modified microelectrode (rectangular black). **d** Repeatability of the PB-modified microelectrode for OLZ sensing

The OLZ sensing performance was evaluated in a 20 µl volume of an undiluted serum sample from a healthy volunteer using the bare and the PB-modified microelectrode. The DPVs were recorded for different concentrations of OLZ-spiked undiluted serum samples. The bare Au microelectrode was unable to differentiate between different OLZ concentrations (Fig. 6a), whereas the PB-modified microelectrode showed a positive dose response with a high background. The linear dose response plot of the PB-modified microelectrode resulted in a sensitivity of 0.14 ± 0.02 µA/cm^2^ nM and an LOD value of 28.6 ± 1.3 nM. The LOD value is well below the clinical threshold.

*PB-modified microelectrode* (*R*^2^ = 0.98; LOD = 27.8 ± 3.9 nM):

4 $$ j_{{\text{anodic peak}}} = \left( {0.14 \pm 0.02 \mu {\text{A}}/{\text{cm}}^{2} {\text{nM}}} \right)\left[ {{\text{OLZ}}} \right] + \left( {28.6 \pm 1.3 \mu {\text{A}}/{\text{cm}}^{2} } \right) $$

The repeatability of the PB-modified microelectrode was tested for 150 nM OLZ-spiked 20 µl undiluted serum samples. In order to reduce the effect of fouling or residuals from the previously measured sample, the modified microelectrode was cleaned between consecutive measurements with PBS solution, followed by DI water cleaning, and then gently dried with fine wipes. At the 5th repetition, the PB-modified microelectrode was able to retain 80.6%, and at the 10th repetition, it was able to retain 60% of the current from the first measurement.

## Conclusions

Point-of-care monitoring of antipsychotics using electrochemical sensors shows great potential for the therapeutic management and treatment of schizophrenia. Modification of microelectrodes with PB enabled OLZ detection in an undiluted finger-pricked serum sample. By further miniaturizing and integrating PB-modified microelectrodes into point-of-care testing devices, the management of schizophrenia treatment can be greatly improved. Our long-term goal is to build the capabilities needed to access complementary chemical information (e.g., from blood). OLZ detection from serum is a critical step in developing sensors for clinical analyses. Importantly, this sensor measurement requires minimal sample pretreatment (only centrifugation to generate the serum), which is in contrast to other approaches that employ additional steps to remove protein, dilute interferents, or adjust pH to facilitate analysis.
